# HIV Risk and Associations of HIV Infection among men who have sex with men in Peri-Urban Cape Town, South Africa

**DOI:** 10.1186/1471-2458-11-766

**Published:** 2011-10-05

**Authors:** Stefan Baral, Earl Burrell, Andrew Scheibe, Ben Brown, Chris Beyrer, Linda-Gail Bekker

**Affiliations:** 1 Department of Epidemiology, Johns Hopkins School of Public Health, Baltimore, MD, USA; 2 Desmond Tutu HIV Foundation, Cape Town, South Africa; 3 School of Medicine, UCLA, Los Angeles, California; 4 Center for Public Health and Human Rights, Johns Hopkins School of Public Health, Baltimore, MD, USA; 5 Institute of Infectious Disease and Molecular Medicine, and Department of Medicine, University of Cape Town, South Africa

## Abstract

**Background:**

The HIV epidemic in Sub Saharan Africa has been traditionally assumed to be driven by high risk heterosexual and vertical transmission. However, there is an increasing body of data highlighting the disproportionate burden of HIV infection among MSM in the generalized HIV epidemics across of Southern Africa. In South Africa specifically, there has been an increase in attention focused on the risk status and preventive needs of MSM both in urban centers and peri-urban townships. The study presented here represents the first evaluation of HIV prevalence and associations of HIV infection among MSM in the peri-urban townships of Cape Town.

**Methods:**

The study consisted of an anonymous probe of 200 men, reporting ever having had sex with another man, recruited through venue-base sampling from January to February, 2009.

**Results:**

Overall, HIV prevalence was 25.5% (n = 51/200). Of these prevalent HIV infections, only 6% of HIV-1 infected MSM were aware of their HIV status (3/50). 0% of men reported always having safe sex as defined by always wearing condoms during sex and using water-based lubricants. Independent associations with HIV infection included inconsistent condom use with male partners (aOR 2.3, 95% CI 1.0-5.4), having been blackmailed (aOR 4.4, 95% CI 1.6-20.2), age over 26 years (aOR 4.2, 95% CI 1.6-10.6), being unemployed (aOR 3.7, 95% CI 1.5-9.3), and rural origin (aOR 6.0, 95% CI 2.2-16.7). Bisexual activity was reported by 17.1% (34/199), and a total of 8% (16/200) reported having a regular female partner. Human rights violations were common with 10.5% (n = 21/200) reporting having been blackmailed and 21.0% (n = 42/200) reporting being afraid to seek health care.

**Conclusions:**

The conclusions from this study include that a there is a high risk and underserved population of MSM in the townships surrounding Cape Town. The high HIV prevalence and high risk sexual practices suggest that prevalence will continue to increase among these men in the context of an otherwise slowing epidemic. These data further highlight the need to better characterize risk factors for HIV prevention and appropriate targeted combination packages of HIV interventions including biomedical, behavioural, and structural approaches to mitigate HIV risk among these men.

## Background

South Africa has a generalized HIV epidemic with an estimated HIV prevalence of 16.9% among adults 15 and older and 13.1% among men aged 15 and older [1]. HIV transmission has been most commonly linked with high risk heterosexual and vertical transmission and consequently this is where the majority of HIV prevention efforts have been targeted. However, across the African continent, the sexual transmission of HIV between men has been increasingly recognized [2]. HIV prevalence studies among men who have sex with men (MSM) have been completed in numerous countries in Southern and Eastern Africa including Malawi, Namibia, Botswana, Tanzania, Uganda, Kenya, and South Africa  [3-5].

Notably, the initial presentation of the HIV epidemic in major urban centers of South Africa was among gay men and other MSM [6]. A study of 250 MSM in Johannesburg in 1983 demonstrated an HIV prevalence of 12.8% with 35.6% infected with syphilis, and another 41.2% positive for Hepatitis B virus antibody [7]. The last few years has seen a resurgence of studies focused on MSM, but there was limited epidemiologic research conducted in the interim three decades. In 2006, a study of rural South African men found that approximately 3.6% of men studied (n = 46/1277) reported a history of having had sex with another man [8]. The adjusted odds ratio for prevalent HIV infections was 3.6 (95% CI 1.0-13.0, p = 0.05) for those reporting same sex practices [8]. One of the first studies published that targeted MSM was completed using respondent driven sampling (RDS) by Lane et al. in Soweto, a peri-urban community south-west of Johannesburg, in 2008 [9,10]. Among 378 MSM, the RDS-weighted HIV prevalence was 13.2% (95% CI 12.4-13.9) with independent associations of prevalent HIV infection being older than 25 (adjusted odds ratio (aOR) 3.8, 95% CI 3.2-4.6), reporting any unprotected receptive anal intercourse (aOR 4.4, 95% CI 3.5-5.7), and having between 6 and 9 male partners in preceding 6 months (aOR 5.7, 95% CI 4.0-8.2). In 2009, an HIV prevalence and risk assessment of 542 MSM attending lesbian, gay, and bisexual (LGBT) venues in urban Cape Town showed an HIV prevalence of 10.4% (56/539). Self-identifying as gay, homosexual, or queer (aOR 4.5, 95% CI 1.0-20.0), and reporting ever having had a sexually transmitted infection (STI) (aOR 4.3, 95% CI: 2.3-8.3) were significantly associated with testing HIV positive [11].

More recently published studies have demonstrated higher HIV prevalence rates including the Johannesburg/eThekwini Men's Study (JEMS) which demonstrated a HIV prevalence rate of 49.5% among 204 MSM in Johannesburg and 27.5% among 81 MSM in Durban [12]. MSM in urban Cape Town also participated in the first multisite randomized controlled trial of antiviral chemoprophylaxis completed [13]. While the HIV prevalence was higher than previous estimates, associations of infection were similar with self-reporting as gay or homosexual (aOR, 8.4; 95% CI, 3.7-19), receptive unprotected anal intercourse (aOR 4.3; 95% CI 2.4-7.6); sex with a person known to be HIV positive (aOR 2.3; 95% CI 1.1-4.9); and a sexually transmitted infection diagnosis (aOR 2.4; 95% CI 1.1-5.2).

South Africa has an HIV epidemic that has disproportionately affected women and girls, especially among those aged 15-29 where prevalence rates have been shown to be significantly higher than their male age counterparts [14]. Fortunately, there is evidence that there have been marginal recent declines in incident HIV infections especially among women and girls resulting in declines in the overall epidemic [15]. Among similarly aged men, HIV prevalence rates have been consistently under 10% HIV prevalence in South Africa [14,15]. While there is significant variability among the estimates of the burden of HIV among MSM in South Africa, when comparing the rates to age-matched men not reporting same-sex practices, the rates are uniformly and significantly higher. Comparisons of prevalence across sampling designs and time frames are always problematic, but this is particularly challenging for hidden and hard-to-reach populations, where behaviours are stigmatized, and for populations for which size estimates are uncertain. All of these potential sources of measurement error pertain to MSM in African settings. Nevertheless, enough data has emerged to suggest significant HIV disease burdens among these men.

The study presented here represents the first evaluation of HIV prevalence among MSM in the peri-urban townships of Cape Town. The study was designed as an epidemiologic probe of MSM in the townships in terms of socio-demographic characteristics, HIV-risk practices, HIV prevalence and associations with being seropositive, and prevalence of human rights violations. The aims of this project were to characterize this population, assess levels of need, and identify barriers to access to HIV preventive services.

## Methods

### Study Sample and Sample Size

Inclusion criteria for this study included being 18 years of age or older, greater Cape Town residence at the time the study was conducted, and reporting a lifetime history of ever having had anal intercourse with another male. Exclusion criteria included having participated in this study previously, unable to orally consent to study participation, and being born female. Sexual orientation/identity, frequency of sexual experience, and previous HIV testing did not preclude study eligibility. Sample size calculations were based on measuring the risk associated with unprotected anal intercourse (UAI). Assuming that UAI increased risk of HIV transmission by approximately 80% with a significance level of 0.05 and a power of 80%, the minimum necessary sample size was 150 men (19). This was rounded up and 200 MSM were recruited for this study. All 200 respondents were enrolled in 24 days at 12 different venues in Cape Town from January to February, 2009.

### Recruitment Strategy

Accrual was completed using a venue-based sampling approach with peer-referral at each venue. Formative work included assessing different MSM-frequented venues including bars, dance clubs, street locations, and social organizations. However, the study team was not able to develop a sampling frame for MSM venues given the dynamic nature of MSM-frequented venues in the townships. As such, the study sample represents a convenience sample rather than a probability-based sample.

Recruitment staff attended these venues and approached men in order to provide brief information about the study and assess eligibility. Additionally, participants were asked to refer their friends/acquaintances who were at the sampling venue at that time. All potential participants in attendance who met aforementioned inclusion and exclusion criteria were consented and then offered participation in the study. All 200 respondents were enrolled in 24 days at 12 different venues in Cape Town from January through February, 2009. Questionnaires were labelled with the venue, date, and a numerical code unique to each participant. This enabled anonymous linking of the results of the survey with HIV-1/2 test results.

Six community members were provided training on how to administer surveys and provide informed consent for all study participants. Study staff were also provided training on best practices in the maintenance of confidentiality and methods to avoid repeat sampling.

### Survey Instrument

After oral informed consent was obtained, study staff trained for this role administered a 45 question survey instrument which included demographics, sexual risk practices, and rights contexts. The instrument did not collect identifiable information from study participants. This instrument has been used in a number of Sub-Saharan African countries with MSM including Malawi, Namibia, Botswana, Lesotho, Nigeria, and Guinea-Bissau. In each setting, there is a formative phase where each question in the instrument is discussed in partnership with community representation as was done in Peri-Urban Cape Town. Transactional sex was defined as whether men reported having traded anal sex with a non-regular partner for money, drugs, food, shelter, or transportation. The entire instrument was also piloted with community representation and modified accordingly [3].

### HIV Testing

HIV-1/2 infection was assessed via oral mucosal transudate specimens collected anonymously from consented participants, and analyzed with the OraQuick^® ^rapid HIV-1/2 antibody test. This testing method has been licensed by the US FDA and has a known sensitivity of 99.1% for oral fluid (compared to 99.7% with serum), and a specificity of 99.6% with oral fluid (compared to 99.9% with serum) [16]. All study staff were trained in accordance with the standardized testing procedures of OraSure^® ^Technologies Inc. No confirmatory test was conducted. OraQuick^® ^HIV-1/2 test results were not diagnostic thus results were not returned to participants. Each participant was provided with a safer-sex kit containing condoms, water-based lubricant, and information on where to obtain free and LGBT-friendly HIV voluntary counselling and testing (VCT), and was strongly encouraged to attend VCT and know their HIV status. After completion of the questionnaire and donation of a salvia sample, participants were reimbursed ZAR 20 (US$ 2.50) for their time.

### Statistical Analysis

Data analysis was performed using STATA 10.0 (STATA Corp, College Station, Texas USA). Bivariate and multivariate logistic regression analyses were used to assess independent predictors of HIV and any cross-sectional association between risk factors and HIV status. Bivariate analyses included two-sample tests for differences in proportions, χ^2 ^tests of independence, and logistic regression assessing the associations between risk factors and HIV status. Backward elimination with a p-value set to 0.1 was used to determine which variables were included in the multivariate model. P-value of 0.1 was chosen to balance the development of a parsimonious model while still including those variables that were of borderline significance. The multivariate logistic regression models included adjustment for factors that were part of an a priori framework of potential associations as well as significant in univariate analyses. In addition, basic demographic characteristics were forced into the model including age and education. Variables that were significantly (p < 0.05) associated with HIV status are reported, accompanied by respective adjusted odds ratios (aOR) and 95% confidence intervals. Because men were recruited by township venue, and in each of the Black and Coloured Townships men were all of the same racial/ethnic group, we did not adjust for race in this analysis, but have presented findings by Township ethnic majority group.

Bisexuality was defined as having at least one male and female sexual partner in the preceding 6 months. A bivariate analysis was conducted using bisexuality as the dependent variable. Factors significantly associated with bisexuality were included in multivariate logistic regression model which also included key demographic characteristics including age, sex, and rural or urban origin, to assess independent associations with bisexual behaviour.

### Ethics Review

The study was approved by the Research Ethics Committee of the University of Cape Town and Institutional Review Board of the Johns Hopkins Bloomberg School of Public Health.

## Results

### Selected Characteristics of study participants

Table 1 demonstrates selected sociodemographic characteristics of MSM sampled in Peri-Urban Cape Town. The mean age was young at 26 (Range 18-58) and a median of 24 with an inter-quartile range of 21 - 29. The men sampled for this study were nearly exclusively born in South Africa with just under half of the sample 46.3%, n = 88/190) originally from rural locales. The majority of men self-reported as being gay or homosexual (77%, n = 154/200) as compared to 18.0% (n = 36/200) self-reporting as bisexual, 4% (n = 8/200) self-reporting as being male to female transgender and only 2 out of 200 men (n = 1%) self-reporting as being heterosexual.

**Table 1:** Selected Characteristics of MSM in Peri-Urban Cape Town in January to February, 2009

Characteristic		Estimate	n/N
Age	Mean/Median (Range)	26.1/24 (18-58)	

Rural Origin		51.5%	103/200

Married		8.0%	16/200

Born in South Africa		97.5%	195/200

Education	Secondary or less	56.0%	112/199

	Tertiary or Vocational School	44.0%	88/199

Employment	Currently employed	60.3%	120/199

Self-Reported Sexual Orientation	Gay/Homosexual	77.0%	154/200
	
	Bisexual	18.0%	36/200
	
	Transgender	4.0%	8/200
	
	Heterosexual	1.0%	2/200

Aware of sexual orientation	Any Family Member	68.5%	137/200
	
	Any Health Care Worker	50%	100/200
	
	Family or Health Care Worker	76.5%	153/200

In last 6 months:	Have injected illegal drugs (IDU)	2.5%	5/200
	
	Found male partner on internet	35.9%	71/198

Self-reported biggest threat to health	HIV	53.6%	105/196
	
	Illegal Drugs	19.4%	38/196
	
	Mental illness	8.7%	17/196
	
	Violence	7.1%	14/196
	
	Sexually Transmitted Infections	5.3%	11/196
	
	Malaria/Tuberculosis	2.0%	4/196

± Not all columns add up due to missing values

Over two-thirds of the men (68.5%, 137/200) had disclosed their sexual orientation to a family member, but only 50%, n = 100/200) had ever disclosed their sexual orientation to a health care worker. Injection drug use (IDU) was uncommon with only 2.5% (n = 5/200) reporting this practice in the last 6 months. Notably, more than one third of the men sampled had used the internet to find a male sexual partner in the preceding 6 months (35.9%, n = 71/198).

### Sexual Practices

The mean number of unique male partners in preceding six months was 4.1 (Range 0-75) with a median of 2 (IQR 0-3). In this sample, 17.7% (n = 35/197) reported more than 5 male partners during this time frame. Far fewer study participants reported female partners with a mean of 0.49 (Range 0-12) in the preceding 6 months as compared to male partners (p < 0.05). During the previous 6 months 17.1% (n = 34/199) reported both male and female sexual partners. Stable partners were defined as a self-reported ongoing relationship such as marriage or having a boyfriend, of the MSM sampled 46% (n = 92/200) were in a stable relationship with only a man, 3.0% (n = 6/200) were in a stable relationship with only a woman. In addition, 3.0% (n = 6/200) were in bisexually concurrent stable relationships with both men and women.

Consistent condom use with all sexual partners was reported by 6.5% (n = 12/184) of participants, though 52.4% reported always wearing condoms with male sexual partners and 39.5% reported always wearing condoms with female sexual partners. Of those reporting commercial lubricant use during anal intercourse, 1.9% (n = 3/155) reported using water-based lubricants as compared to 98.1% who reported using petroleum based products (n = 152/155). An additional 18.6% (n = 37/199) reported using saliva as a lubricant for anal intercourse and 3.5% (n = 7/199) reported no lubricant use. Overall, 0% of men reported consistent condom and water-based lubricant use.

### Bisexual Activity and Concurrency

Active bisexual practices were defined as having both at least one male and female sexual partner in the preceding 6 months [17]. Bisexual activity was reported by 17.1% (34/199), and a total of 8% (16/200) reported having a regular female partner. In bivariate analyses, bisexual practices were significantly associated with always wearing condoms (OR 11.7, 95% CI 3.3-41.8, p < 0.01), not having disclosed sexual orientation to either family or a health care worker (OR 0.32, 95% CI 0.15-0.72, p < 0.05), having received money for casual sex with a man (OR 4.5, 95% CI 1.8-11.3, p < 0.05) and a likelihood of prevalent HIV infection (OR 0.33, 95% CI 0.11-1.0, p < 0.05). In the multivariate model including all variables that were significant in bivariate analyses along with key demographic characteristics including age, sex, and rural or urban origin, independent associations with active bisexuality were always wearing condoms (aOR 11.8, 95% CI 2.8-49.3), not having disclosed sexual orientation to immediate family (aOR 0.39, 95% CI 0.16-0.96), having received money for transactional sex (aOR 10.9, 95% CI 3.3-34.8), and being less likely to be HIV positive (aOR 0.28, 95% CI 0.08-0.9).

### HIV Prevalence

Overall, HIV prevalence was 25.5% (n = 51/200). There was a significant difference in HIV prevalence among MSM accrued in the Black townships (37.3%, n = 38/102) as compared to those accrued at venues in the Coloured townships (12.5%, n = 11/88) (p < 0.01). Only 6% of HIV-1 infected MSM were aware of their HIV status (3/50).

### Associations with HIV Infection

Significant bivariate associations with prevalent HIV infection were being older than 26 years old years old (OR 2.0, 95% CI 1.05-3.8, p < 0.05); having secondary-level education or less (OR 0.29, 95% CI 0.11-0.76, p < 0.01); no reported unprotected anal intercourse (OR 0.52, 95% CI 0.27-1.01, p = 0.054); reporting >5 male partners in the previous 6 months (OR 2.8, 95%CI 1.31-6.1, p < 0.01); having received money or goods for anal intercourse with another man (OR 2.9, 95% CI 1.2-6.9, p < 0.05, having been blackmailed (OR 2.44, 95% CI p = 0.054), being from a city (OR 0.42, 95% CI 0.23-0.77, p < 0.01), and reported ever having been raped by a man (OR 3.45, 95% CI 1.39-8.54, p < 0.01). The multivariate model (Table 2) used prevalent HIV infections as the outcome and included demographic characteristics age, employment status, rural origin, and the following variables: not always wearing condoms with men, having received money or goods for transactional sex, having been blackmailed or raped, higher numbers of male partners, unemployment, lower age and education level. Independent associations with HIV infection included inconsistent condom use with male partners (aOR 2.3, 95% CI 1.0-5.4), having been blackmailed (aOR 4.4, 95% CI 1.6-20.2), age over 26 years (aOR 4.2, 95% CI 1.6-10.6), being unemployed (aOR 3.7, 95% CI 1.5-9.3), and rural origin (aOR 6.0, 95% CI 2.2-16.7). (Table 2).

**Table 2:** Univariate and Multivariate Logistic Regression to Characterize Associations with HIV Status among MSM in Peri-Urban Cape Town

Characteristic	Total	HIV Positive		Univariate	Multivariate
	**n (%)**	**Yes n (%)**	**No n (%)**	**OR (95% CI)**	**aOR (95% CI)**

Not always wearing condoms with men	88 (47.6%)	18 (20.5%)	70 (79.5%)	1.9 (1.0-3.7), *	2.3 (1.0-5.4),**

Received money for Transactional Sex	24 (12.0%)	11 (45.8%)	13 (54.2%)	2.9 (1.2-6.9),*	NS

Having been blackmailed	21 (10.5%)	9 (42.9%)	12 (57.1%)	2.4 (1.0-6.2) (p = 0.06)	4,4 (1.3-15.4), *

Having been raped	22 (11.0%)	11 (50.0%)	11 (50.0%)	3.5 (1.4-8.5),**	5.7 (1.6-20.2) **

Higher number of male partners (>5)	35(17.7%)	15 (42.9%)	20 (57.1%)	2.8.(1.3-6.1), **	NS

More than 26 years old	77 (38.5%)	26 (33.8%)	51 (66.2%)	2.0 (1.1 - 3.8), *	4.2 (1.6-10.6),**

Unemployment	79 (39.7%)	27 (34.2%)	52 (65.8%)	2.2 (1.1-4.2), *	3.7 (1.5-9.3),**

Less Educated	20 (10.0%)	10 (50.0%)	10 (50.0%)	3.4 (1.3.-8.8), *	NS

Rural Origin	102 (53.6%)	64 (62.7%)	38 (37.3)%)	4.1 (2.0-8.8),**	6.0 ( 2.2-16.7), **

± Not all columns add up due to missing values

*p < 0.05

**p < 0.01

NS - Not Significant

### Human Rights Violations

This study assessed prevalence of human rights violations among MSM in peri-Urban Cape Town. 5% (n = 10/200) of the study sample reported being denied either housing or health care based on their sexuality. In addition, 10.5% (n = 21/200) reported having been blackmailed and 8.0% (n = 16/200) reported having been beaten by police or a government official based on their sexuality. 21.0% (n = 42/200) of the men included in the study reported being afraid to seek health care services based on their sexuality. 11.0% of the study participants (n = 22/200) reported ever having been raped by a man. Finally, 9.1% (n = 18/199) were afraid to walk in their own community because of their sexuality. Overall, 24.5% (n = 49/200) of the men sampled reported at least one human rights violation.

## Discussion

This study presents the first data describing burden of HIV and associations of prevalent HIV infection among MSM in the Peri-urban townships surrounding Cape Town. As with the majority of studies of MSM in Africa, the study sample tended to be young, with a mean age of 25. However, the study demonstrated a significant and disproportionate burden of disease among these young men [15]. While analytic comparisons using direct or indirect age standardization have not yet been done to the knowledge of the authors, these data along with other recent descriptions of predominantly young MSM suggest that HIV risks and rates are consistently high in Sub-Saharan Africa [2].

The estimates of HIV prevalence observed here were significantly higher than a recent HIV prevalence study of MSM in urban Cape Town [11]. In that study, HIV prevalence was found to be 10.6% (n = 57/538) as compared to 25.5% (n = 51/200) observed in this study (p < 0.05) (23). While prevalence in the townships was higher, there were not notable differences observed in levels of sexual risk practices. This apparent contradiction may be due to varying structural drivers of HIV-related risk, including social determinants of health and human rights violations. Network level factors, including the background population HIV prevalence, may also play a role.

Human rights violations in this sample of MSM were common, with nearly a quarter of the participants reporting at least one rights violation. This study demonstrated the public health outcomes of these rights violations, including a higher prevalence of HIV among those MSM who reported blackmail, both in bivariate and multivariate analyses. The experience of these men suggests that there is a disconnect between legally mandated protections in the constitution and daily life in for MSM in the townships studied here [18,19]. The implications for combination HIV prevention interventions are significant as these results suggest that human rights violations would interfere with the ability to identify, accrue, and prospectively retain MSM.

This study found lower rates of active bisexuality and bisexual concurrency among MSM than in other countries in the region. This may be related to better social and legal contexts for gay men in South Africa as compared to other South African Development Community (SADC) countries (3;14). Understanding the associations of bisexual practices and bisexually concurrent partnerships can be help inform programs targeting behavior change and partner reduction that traditionally target exclusively heterosexual multiple concurrent partnerships. While qualitative studies are needed to further characterize drivers of bisexual practices, it is conceivable that men who feel more comfortable in same-sex relationships will be less likely to have female partners. Encouragingly, Black and Coloured South African MSM practicing active bisexuality reported higher rates of condom use and were less likely to be infected with HIV then were men who reported only sex with men. The observation of lower HIV risks among bisexually active MSM has been seen in a number of studies of MSM in the African context (10;14;15;26).

There were numerous limitations with the study design limiting the generalizability of the results to all MSM in South Africa. Primarily, this study was cross-sectional which prevents any characterization of causality between the associations described here and incident HIV infections. In addition, recruitment occurred at social venues for gay men and other MSM in townships which limits the generalizability of the findings to MSM who frequent these social venues. While the men were accrued using venue-based sampling, there was no sampling frame of all venues characterized highlighting that these results are not probability estimates of risk and burden of disease. Strategies that may be feasible to obtain probability estimates or unbiased non-probability estimates include venue-day-time sampling or respondent driven sampling (RDS). Gay venues in the townships tend not be brick and mortar bars, as seen in urban Cape Town; rather they are more clandestine venues that shift location. While this would limit time-location sampling such as has been done successfully with MSM in Thailand [20], RDS is an option to generate unbiased parameter estimates approximating population-based estimates of disease burden and predictors of disease among MSM in these contexts [21]. Population-based HIV surveillance strategies such as demographic health surveys are not well suited for MSM in Africa given that these tend to be household based surveys. If administered in a household, same-sex practices may be underreported. The aforementioned active and targeted recruitment methods will likely be required to accrue MSM in settings where these practices remain stigmatized and even criminalized.

HIV risk-related practices among these men were very common, with relatively low reported rates of both always wearing condoms with all sexual partners, and using water-based lubricants during sex. Indeed, 0 men reported regularly practicing safe sex with either men or women as defined above. There were numerous individual-level associations with HIV risk that were expected based on previous studies including not wearing condoms, higher numbers of male partners, and having received money for transactional sex [3,22,23]. However, as described there were also structural drivers of HIV including rape and blackmail. In response to these multilevel risks for HIV infection among MSM, it is vital to explore and evaluate combination HIV prevention interventions (CHPI) for MSM including behavioural, biomedical, and structural interventions [24].

## Conclusions

While there were limitations in the methodology of the study, it is appropriate to conclude that there are MSM living in peri-urban Cape Town townships that are at significant risk of HIV infection. With the emergence of new biomedical strategies such as oral and topical antiviral chemoprophylaxis to prevent HIV infection, these men should be considered as a high risk population that population that are appropriate for inclusion into large scale prevention trials. However, to ensure appropriate risk identification accrual and retention of these men, structural interventions such as health sector interventions and building social capital should be included in the package of combination HIV preventive interventions.

## Competing interests

The authors declare that they have no competing interests.

## Authors' contributions

SB, EB, LGB, CB conceived of the study and methods section. EB was the primary implementer of this work under the supervision of LGB. EB, AS, SB, BB, developed the analytic plan and SB and EB led the analysis. The manuscript was written as a collaboration between SB, CB, EB, BB, and AS. LGB and CB provided ongoing supervision throughout the life of the project. All authors have seen and approved the final version of this manuscript.

## Pre-publication history

The pre-publication history for this paper can be accessed here:

http://www.biomedcentral.com/1471-2458/11/766/prepub
